# Country Immunization Information System Assessments — Kenya, 2015 and Ghana, 2016

**DOI:** 10.15585/mmwr.mm6644a5

**Published:** 2017-11-10

**Authors:** Colleen Scott, Kristie E. N. Clarke, Jan Grevendonk, Samantha B. Dolan, Hussein Osman Ahmed, Peter Kamau, Peter Aswani Ademba, Lynda Osadebe, George Bonsu, Joseph Opare, Stanley Diamenu, Gregory Amenuvegbe, Pamela Quaye, Fred Osei-Sarpong, Francis Abotsi, Joseph Dwomor Ankrah, Adam MacNeil

**Affiliations:** ^1^Global Immunization Division, CDC; ^2^World Health Organization, Geneva, Switzerland; ^3^Kenya Immunization Information System team; ^4^Ghana Immunization Information System team.

The collection, analysis, and use of data to measure and improve immunization program performance are priorities for the World Health Organization (WHO), global partners, and national immunization programs (NIPs). High quality data are essential for evidence-based decision-making to support successful NIPs. Consistent recording and reporting practices, optimal access to and use of health information systems, and rigorous interpretation and use of data for decision-making are characteristics of high-quality immunization information systems. In 2015 and 2016, immunization information system assessments (IISAs) were conducted in Kenya and Ghana using a new WHO and CDC assessment methodology designed to identify root causes of immunization data quality problems and facilitate development of plans for improvement. Data quality challenges common to both countries included low confidence in facility-level target population data (Kenya = 50%, Ghana = 53%) and poor data concordance between child registers and facility tally sheets (Kenya = 0%, Ghana = 3%). In Kenya, systemic challenges included limited supportive supervision and lack of resources to access electronic reporting systems; in Ghana, challenges included a poorly defined subdistrict administrative level. Data quality improvement plans (DQIPs) based on assessment findings are being implemented in both countries. IISAs can help countries identify and address root causes of poor immunization data to provide a stronger evidence base for future investments in immunization programs.

In 2001, WHO developed a methodology, the Data Quality Audit (*1*) to be used in lower- and middle-income countries to assess NIP administrative vaccination coverage data quality (*2*,*3*). WHO adapted this methodology for NIPs as a self-assessment tool, the Data Quality Self-Assessment (*4*). However, these methodologies focused on data validation and often missed underlying systemic issues, sometimes resulting in recommendations that were not actionable, not implemented, or that had little impact (*5*,*6*). In 2014, WHO and CDC collaborated to develop updated guidance for IISAs. Designed to be adaptable to a specific country context, the IISA guidance consists of four modules (Box). Modules are designed to identify the root causes of data quality problems and inform the development of actionable DQIPs.

Box **Immunization information system assessment modules**

**Module 1: Desk Review**

- Review of systems, processes, governance, and workforce to create an immunization data flow diagram.
- Support from a checklist and implemented through individual and focus group interviews.
- A systematic review of forms, tools, and the reports of previous assessments is performed to identify redundant tools and follow up any actions taken on previous recommendations.

**Module 2: National Data Review**

- Evaluation of the completeness, internal consistency, trends, and external consistency of national administrative vaccination coverage data through triangulation with external sources following a defined protocol.

**Module 3: Field Data Collection**

- Field teams administer a qualitative questionnaire and triangulate multiple sources of immunization data in a purposive sample of geographic regions, subnational sites, and health facilities.
- Team members are assigned a thematic area on which to focus observations during site visits.
- Topics include the following:
  - Recording and data verification
  - Data reporting, analysis
  - Denominator
  - Workforce, training, and human resources

**Module 4: Data Quality Improvement Plan (DQIP) Development**

- Debrief and review of all data and information gathered in the prior three modules.
- Develop a plan through root cause discovery using an established framework with engagement of stakeholders.

The first IISA was conducted in Kenya in 2015. The desk review and national data review modules were performed remotely over a 3-month period using data and documents gathered by the Kenya Ministry of Health; the reviews were finalized 2 months before fieldwork began (Figure). Field questionnaires were refined using desk review findings and pilot testing. Teams collected data from four counties, eight subcounties, and 16 health facilities over a 5-day period. The DQIP was finalized 6.5 months after conclusion of the fieldwork.

**FIGURE:**
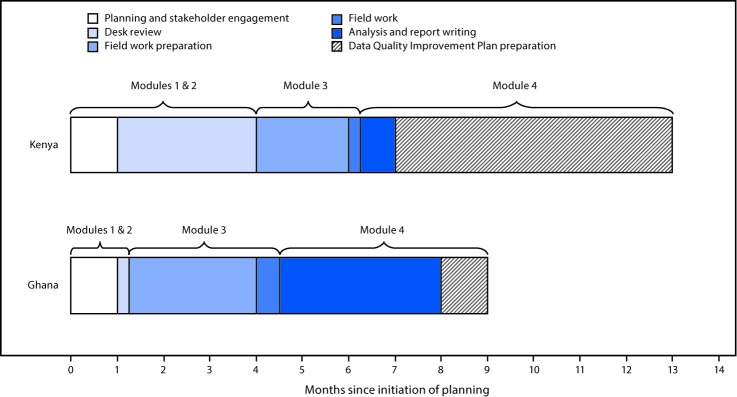
Timeline of key steps* in immunization information system assessments^†^ — Kenya, 2015 and Ghana, 2016 * Indicates time between initiation of key steps rather than time of continuous work on each step; work on each module had to fit within the national immunization program calendar. ^†^ Module 1 = desk review; Module 2 = national data review; Module 3 = field data collection; Module 4 = Data Quality Improvement Plan development.

An IISA was conducted in Ghana during 2016; modules were adapted to suit country needs. The desk review and national data review modules were conducted collaboratively by the Ghana Ministry of Health, WHO, and CDC during a 3-day in-country meeting 2.5 months before commencement of fieldwork. Participants were divided into two teams; one created a detailed description of the immunization data system, and the other analyzed immunization data trends and selected field assessment sites. After piloting the questionnaires, field teams visited four regions, eight districts, 14 subdistricts, and 34 health facilities over 7 days. Teams conducted initial analyses to create region-specific presentations for the debriefing. The DQIP was finalized 4.5 months after completion of the fieldwork.

In both countries, four field data collection teams were deployed for the IISA, each composed of three to four members, including national and subnational ministry of health and NIP officials and one partner (WHO or CDC) representative. Subnational staff members evaluated sites outside their jurisdiction. Purposive sampling was used to select diverse sites, accounting for setting, population density, and vaccination coverage. Field teams used standardized questionnaires to gather information on immunization data practices and challenges. To assess concordance among data sources, teams compared aggregate totals of administered third doses of diphtheria and tetanus toxoid and pertussis (DTP3) vaccine and oral poliovirus (OPV3) vaccine among different facility data collection tools (tally sheets, monthly reports, and child registers). These totals were compared with data at higher administrative levels. After data analysis was finalized, a DQIP was developed.

Assessment results indicated a range of performance across indicators in Kenya and Ghana (Table). Staff members in 10 of 16 health facilities in Kenya and 23 of 34 in Ghana reported meeting monthly to discuss vaccine administration data. However, only five health facilities in Kenya and 14 in Ghana displayed these data using an updated monitoring chart. Staff members in half of facilities (Kenya = 50%, Ghana = 53%) reported that monthly targets for immunization of children aged <1 year were not accurate; targets were felt to be too high or too low compared with the actual population size. Reasons cited by staff members for concerns about target population sizes were similar across sites, including population migration and clients crossing between ill-defined health facility catchment areas. Staff members at most facilities (Kenya = 81%, Ghana = 100%) reported needing additional training in at least one of the following immunization-data–quality domains: record-keeping, reporting, analysis, and use for action.

**TABLE:** Vaccine administration data concordance* and selected data quality and data use indicators, by country— Kenya immunization information system assessment (IISA), 2015 and Ghana IISA, 2016

Selected data quality and data use indicators from IISA	No. subnational sites (%)	
	Kenya, n = 8	Ghana, n = 16
Subnational level		
Concordance between received facility monthly report and subnational database	5 (63)	4 (25)
**Health facility data quality and use indicators**	**No. facilities (%)**	
	**Kenya, n = 16**	**Ghana, n = 34**
Concordance between child vaccination register and facility vaccination tally sheets	0 (0)^†^	1 (3)
Concordance between facility monthly report and facility vaccination tally sheets	5 (31)	13 (38)
Staff members meet at least monthly to discuss immunization data	10 (63)	23 (68)
Up-to-date, properly filled immunization monitoring chart	5 (31)	14 (41)
Staff members felt they need more training in at least one domain of immunization data management	13 (81)	34 (100)
Staff members felt their monthly target population for immunization was not accurate^§^	8 (50)	18 (53)

* Defined as 100% concordance for both the third dose of oral poliovirus vaccine (OPV3) and the third dose of diphtheria and tetanus toxoids and pertussis vaccine (DTP3) over all months compared.

^†^ Field team compared tally sheet and register data at 15 of 16 facilities visited in Kenya.

^§^ Targets were thought to be too high or too low compared with actual population size observed by staff members.

In Kenya, concordance was higher between data reported at the subcounty and health facility levels (63%) than between different data sources within the health facility (0%–31%); in Ghana, concordance was poor between subdistrict and health facility data (25%). In both countries, concordance between immunization tally sheets and child registers at health facilities was low (Kenya = 0%, Ghana = 3%) (Table). Root causes of data quality challenges reported by staff members in Kenya include redundant data collection tools, lack of transportation, limited supportive supervision, and lack of airtime or internet access for electronic data reporting. In Ghana, the subdistrict level is responsible for providing supportive supervision to assigned health facilities. However, subdistrict staff members are co-located within designated health facilities; one set of staff members are responsible for all operations within their own facility as well as subdistrict supervisory activities. Root causes of data quality challenges noted by staff members in Ghana include poorly defined roles of subdistrict staff members and a lack of training on supportive supervision, data management, and interpretation. In contrast, district staff members in Ghana demonstrated proficiency in data analysis, use, and interpretation, based on field team observations of vaccination rate monitoring charts and responses to interview questions on calculation of key indicators.

## Discussion

In addition to identifying opportunities to improve NIP vaccination data quality in each country, the updated approach described here for assessing immunization data quality and developing a plan for improvement in Kenya and Ghana can inform future IISAs. Fieldwork was rapid in both countries; however, scheduling all the steps of an IISA in a condensed period can be challenging because of multiple NIP priorities and activities. One year from initiation of planning to consensus on a DQIP might be a realistic timeframe for many countries. Partner engagement and planning should begin at least 5 months before the projected start of fieldwork. The desk review might vary in duration depending upon the amount of information included, size of the team reviewing, and whether the review is done remotely or in-country. Additional time should be allotted for special circumstances such as political instability or the need for document translation.

The experiences in Kenya and Ghana illustrate that the desk review and national data review modules can be adapted by countries under flexible IISA guidelines. More expedient implementation of the two modules was accomplished in Ghana by working in-country with the Ghana Ministry of Health and partners. Regardless of where reviews are conducted, ministry of health and in-country partners are necessary for compiling the required data and documents. For fieldwork, three to four member teams were sufficient for data collection, yet manageable for facilities. Diverse field teams composed of national, subnational, and partner staff members incorporated multiple viewpoints into findings. Assigning subnational staff members to geographical subunits outside their jurisdiction reduced the potential for bias and provided staff members with a range of perspectives.

Various root causes of data quality challenges were identified. In both Kenya and Ghana, data in health facility registers were incomplete and demonstrated low concordance with other data sources. Other challenges included a low level of confidence in target population data, self-identified need for facility staff member training, and infrequent analysis and use of immunization data. Triangulation of data identified stronger subnational data concordance in Kenya, whereas Ghana had administrative and training support challenges at the subdistrict level.

The findings in this report are subject to at least two limitations. First, findings are not nationally representative, which could have resulted in over- or underestimation of the concordance of vaccination event data between data collection tools and administrative levels. Second, this report describes the data from two countries; because each country is unique, these findings might not be generalizable to other contexts.

Importantly, IISA guidance emphasizes following up all findings with an evidence-based, feasible DQIP developed collaboratively to fit within existing ministry of health and NIP timelines. Concrete actions have been taken based on the findings of the IISAs described. In Kenya, national and county target-setting workshops were convened; as a result, the DQIP was integrated into Gavi, the Vaccine Alliance health systems, strengthening support to 17 selected counties. In Ghana, pilot changes are being made to improve the managerial and supervisory skills of subdistrict staff members. In addition, data quality content is being incorporated into preprofessional coursework for health professional studies as well as continuing education for current staff members. In this way, the updated IISA guidance and its focus on data for action is providing an impetus for long-term change. Ultimately, higher quality immunization data provide better evidence for subsequent investments and interventions related to immunization programs, vaccine preventable disease surveillance, and outbreak response.

**Summary**

**What is already known about this topic?**

The availability, quality, and use of immunization data are widely considered to form the foundation of successful national immunization programs. Lower- and middle-income countries have used systematic methods for the assessment of administrative immunization data quality since 2001, when the World Health Organization (WHO) developed the Data Quality Audit methodology. WHO adapted this methodology for use by national programs as a self-assessment tool, the Data Quality Self-Assessment. This methodology was further refined by WHO and CDC in 2014 as an immunization information system assessment (IISA).

**What is added by this report?**

Findings of immunization information system assessments in Kenya and Ghana identified some common challenges, such as incompleteness of the facility child register, low confidence in target population data, and infrequent analysis and use of data at the facility level. The assessments also examined larger systemic challenges that could explain the root causes of these problems, such as a poorly defined subdistrict administrative level in Ghana and need for training on data quality and data use among facility staff in both countries.

**What are the implications for public health practice?**

The experience gained from implementing assessments using updated IISA guidance in Kenya and Ghana provides an opportunity to inform other countries interested in best practices for assessing their data quality and creating actionable data quality improvement plans. Data quality improvement is important to provide the most accurate and actionable evidence base for future decision-making and investments in immunization programs. This review provides best practice experiences and recommendations for countries to use an IISA to assess data quality from national administrative structure down to the facility level. This methodology also meets the requirements for use by Gavi, the Vaccine Alliance, for monitoring national immunization data quality at a minimum interval of every 5 years in conjunction with funding decisions.
